# The spindle position checkpoint in budding yeast: the motherly care of MEN

**DOI:** 10.1186/1747-1028-1-2

**Published:** 2006-04-03

**Authors:** Simonetta Piatti, Marianna Venturetti, Elena Chiroli, Roberta Fraschini

**Affiliations:** 1 Dipartimento di Biotecnologie e Bioscienze, Universita' di Milano-Bicocca, Piazza della Scienza 2, 20126 Milano, Italy

## Abstract

Mitotic exit and cytokinesis must be tightly coupled to nuclear division both in time and space in order to preserve genome stability and to ensure that daughter cells inherit the right set of chromosomes after cell division. This is achieved in budding yeast through control over a signal transduction cascade, the mitotic exit network (MEN), which is required for mitotic CDK inactivation in telophase and for cytokinesis. Current models of MEN activation emphasize on the bud as the place where most control is exerted. This review focuses on recent data that instead point to the mother cell as being the residence of key regulators of late mitotic events.

## Background

In polarized cells alignment of the mitotic spindle respective to the polarity axis is crucial for maintaining the correct ploidy from one generation to the next. Many cell types deal with this problem by building the cleavage furrow perpendicularly to the spindle and equidistant to the spindle poles [1,2]. In contrast, *S. cerevisiae *(budding yeast) sets up the constriction between mother and daughter cell, called bud neck, already at the G1/S transition concomitant with bud emergence, thus choosing ahead of time the position where cytokinesis will take place. Therefore, it is essential that the spindle be correctly aligned with respect to the mother-bud axis before cytokinesis takes place. A surveillance mechanism called spindle position checkpoint is in charge of responding to spindle misalignment by delaying mitotic exit and cytokinesis, thus providing the time necessary to correct the defects [3].

**Figure 1:**
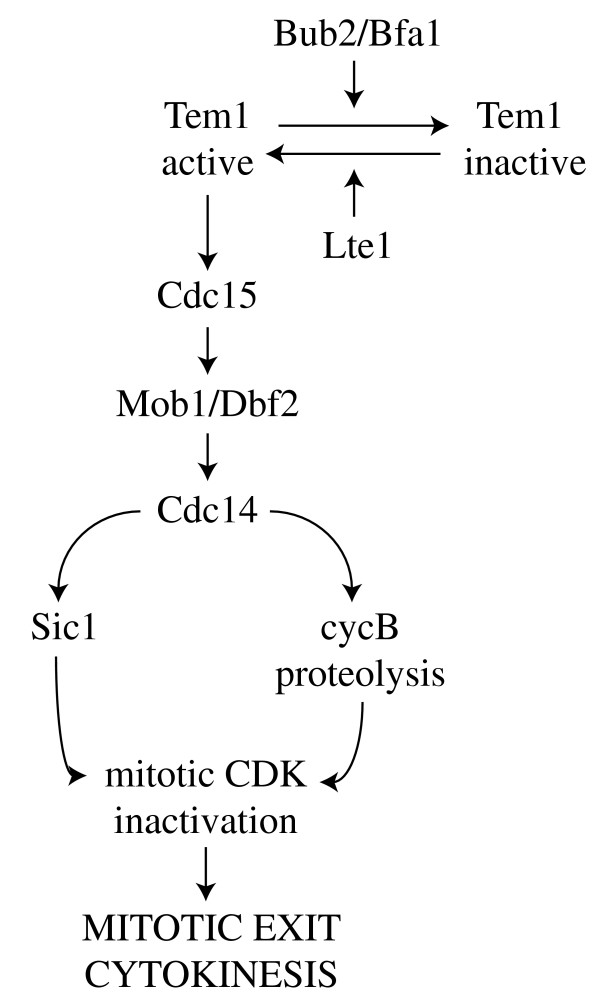
The mitotic exit network.

In all eukaryotes mitotic exit takes place when mitotic cyclin-dependent kinases (CDKs) are inactivated, a task that is usually fulfilled by cyclin proteolysis. Mitotic CDKs inactivation is in turn necessary for spindle disassembly, licensing of replication origins and cytokinesis. The protein phosphatase Cdc14 is key to this process in budding yeast, where it promotes mitotic exit by turning on cyclin proteolysis and by activating the cyclin B-CDK inhibitor Sic1 [4]. Cdc14 is kept inactive throughout most of the cell cycle, anchored in the nucleolus by tight binding to the Net1/Cfi1 inhibitor [5,6]. Cdc14 is partially released into the nucleoplasm at the metaphase to anaphase transition by the FEAR (Cdc fourteen early anaphase release) pathway, whereas the MEN (mitotic exit network) drives its full release also into the cytoplasm later in anaphase, thus allowing it to dephosphorylate its targets (Fig. 1). While the FEAR is dispensable for mitotic exit, the MEN is absolutely required for this process. In case of spindle mispositioning Cdc14 is maintained sequestered in the nucleolus, thereby preventing mitotic exit [7-9].

The MEN is a signal transduction cascade that includes several protein kinases, such as Cdc5 (polo-like kinase), Cdc15, Dbf2 and its associated activator Mob1 (Fig. 1). A similarly organized pathway, the septation initiation network (SIN), promotes cytokinesis in fission yeast [10,11]. Most MEN components are localized during mitosis at microtubule organizing centers, called spindle pole bodies (SPBs) in yeast, whereas they can also be found at the septum just before cytokinesis. SPB localization of MEN components correlates with MEN activation, and mutations that disrupt this localization, like *nud1*, lead to a telophase arrest [12,13]. Interestingly, components of the fission yeast SIN, which comprises proteins homologous to those of the MEN, display a similar localization pattern. The G-protein Tem1 (Spg1 in fission yeast) triggers MEN activation upstream of the aforementioned components by activating the Cdc15 kinase [5,14] and is the direct target of the spindle position checkpoint [10,11]. Its activity is finely tuned by positive (Lte1) and negative (Bub2, Bfa1 and Kin4) regulators (Fig. 1), which all contribute to coupling correctly oriented nuclear division with mitotic exit. This review will focus on the interplay between these regulators during activation of the spindle position checkpoint and on the role of their subcellular localization in controlling mitotic exit.

## Tem1 regulation: GTP binding, GTP hydrolysis or effector exclusion?

Tem1 is a small Ras-like GTPase, supposedly active in the GTP-bound form. However, mutations expected to cause hyperactivation of Tem1, on the basis of their effects on Ras proteins [15], either have no effect or cause a telophase arrest at high temperatures [14]. This temperature-sensitive phenotype is recessive, suggesting that it is due to loss of Tem1 function. In addition, whereas overproduction of wild type Tem1 is sufficient to bypass the checkpoint arrest induced by microtubule depolymerizing drugs [16], overproduced Tem1^Q79R ^and Tem1^Q79K ^(mimicking the dominant active variants Q61R and Q61K of N-Ras) do not (our unpublished data). Thus, a formal proof that Tem1 is active when bound to GTP still awaits experimental support. So far, the best evidence for such a model comes from studies in fission yeast, where the GTP-bound form of Spg1 interacts far more efficiently with the effector kinase Cdc7 (the orthologue of budding Cdc15) than its GDP-bound form [17].

By analogy with other Ras-like GTPases, Tem1 was originally thought to be regulated by the activity of GTPase-activating proteins (GAPs), which decrease the amount of active GTP-bound form by stimulating GTP hydrolysis. On the other hand, guanine-nucleotide exchange factors (GEFs) would promote the conversion of GDP- into active GTP-bound state. Indeed, proteins that might fulfil these two functions have been found in budding yeast, where Bub2 has a domain similar to GAPs, whereas Lte1 has a putative Ras GEF domain, consistent with their role in Tem1 inhibition and activation, respectively [10,11]. Accordingly, Bub2 shows *in vitro *GAP activity on Tem1, but only in combination with Bfa1 [18,19], which forms a complex with Bub2 and mediates its interaction with Tem1 [20-22]. Surprisingly, Bfa1 alone is able to lock Tem1 in the GTP-bound state [18,19]. This activity is reminiscent of guanine nucleotide dissociation inhibitors (GDIs). However Bfa1, like its fission yeast orthologue Byr4, seems unable to prevent GDP release from Tem1, which takes place at extremely high rates [19,23]. The observation that Bfa1 inhibits both dissociation and hydrolysis of GTP raises a paradox and again questions whether the GTP-bound form corresponds to active Tem1. In fact, not only is Bfa1 required, along with Bub2, for inhibiting Tem1 upon activation of the spindle position checkpoint [16,24,25], but it can prevent mitotic exit independently of Bub2 when overexpressed [21,25,26]. It is therefore possible that GTP hydrolysis, rather than GTP binding, is responsible for Tem1 activation. Alternatively, high levels of Bfa1 might inhibit MEN activation by precluding the access of the Cdc15 kinase to the Tem1 effector site [21]. This may take place through a short region of similarity between the fission yeast orthologues of Bfa1 and Cdc15 (Byr4 and Cdc7, respectively), which might hence compete for Spg1 binding [27].

Recent data have challenged further the role of Bub2/Bfa1 GAP activity in Tem1 inhibition. In particular, a myc-tagged version of Bub2, which does not display *in vitro *GAP activity towards Tem1 when combined with Bfa1, is perfectly checkpoint proficient. This implies that the Bub2-myc9/Bfa1 complex (and by extension wild-type Bub2/Bfa1) might inhibit Tem1 through an unanticipated mechanism that does not involve GTP hydrolysis and release [18]. Conversely, a mutation in the catalytic arginine of Bub2 (R85A) abolishes Bub2/Bfa1 GAP activity on Tem1 and is checkpoint-deficient. However, in this case the checkpoint defect might be attributable to the inability of the *bub2*^*R*85*A *^mutant to recruit Bfa1 at SPBs rather than to loss of GAP activity [18]. It should be noted that Tem1 displays relatively high intrinsic GTPase activity (K_hyd_~0.1 min^-1^) and GTP dissociation rate (K_d_~0.1 min^-1^) compared to other Ras-like GTPases [19]. Therefore, accelerating its transition towards a GDP-bound state might not help much in downregulating Tem1. In this regard it is also worth mentioning that a nearly hundred fold excess of Bub2 with respect to Bfa1 is required for a modest stimulation of Tem1 GTPase activity, whereas roughly equimolar amounts of Bfa1 are sufficient to stabilize Tem1 in the GTP-bound form *in vitro *[18,19]. So far, the detailed stoichiometry of the Bub2/Bfa1 complex is not known. In addition, the rates of Tem1 catalysis and nucleotide association/dissociation might be influenced *in vivo *by other players, especially at SPBs. Thus, whether inhibition of Tem1 by Bub2/Bfa1 is achieved by switches in nucleotide binding or by other mechanisms remains an open question.

Among GTPases, Tem1 is peculiar in that it shows an intrinsically high rate of GDP dissociation (K_d_~0.1 min^-1 ^at 13°C, while the off-rate at 30°C is too fast to be measured [19]). In addition, it is able to load GTP efficiently on its own. Consequently, a GEF might not be required for Tem1 activation. The fact that deletion of *LTE1*, the putative GEF, is not lethal but simply confers cold-sensitivity for growth (unlike *TEM1 *deletion) is consistent with this idea [14,28]. Recently, the GEF domain of Lte1 has been shown to be dispensable for viability even at low temperatures, suggesting that Tem1 activation by Lte1 involves a GEF-independent mechanism [29]. One possibility is regulation of Tem1 localization, as Lte1 was shown to be required for preferential Tem1 accumulation on the bud-directed SPB during anaphase (see below, [30]).

## Subcellular localization of Tem1 regulators and control over mitotic exit

**Figure 2:**
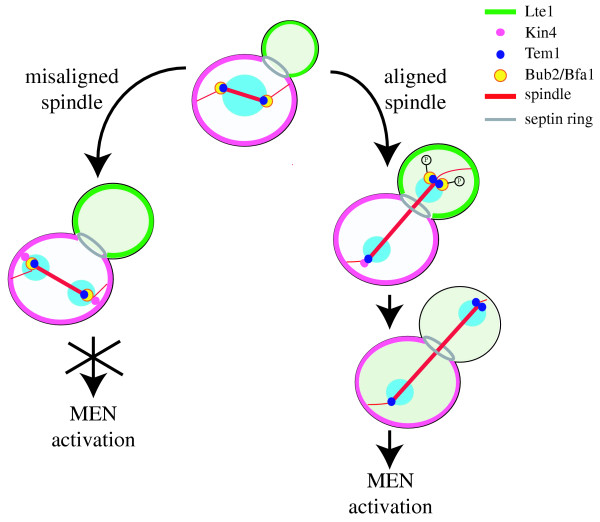
**A model for control over mitotic exit by the spindle position checkpoint. **See text for details. In the presence of misaligned spindle Kin4 and the Bub2/Bfa1 complex are retained at both SPBs and prevent mitotic exit by inhibiting Tem1. When the spindle is correctly positioned, passage of one SPB through the bud neck leads to disappearance of Bub2/Bfa1 from the mother cell SPB and to the exposure of the pool of Tem1 located on the daughter cell SPB to its activator Lte1. At the same time both Tem1 and the Bub2/Bfa1 complex get concentrated on the bud-directed SPB. During this stage the polo kinase Cdc5 phosphorylates Bub2/Bfa1, although at the moment it is unclear where in the cell this regulation takes place. In late anaphase Bub2/Bfa1 disappears also from the daughter cell SPB, whereas Kin4 is removed from the mother-bound SPB. Concomitantly, Lte1 diffuses into the cytoplasm of both mother and daughter cell. Probably, all these events contribute to promote MEN activation. Kin4 localization at the bud neck in late anaphase is not depicted.

The localization of Tem1 and its regulators is tightly controlled during the cell cycle (Fig. 2). Tem1, which is present on the single SPB in G1, gets distributed with roughly equal amounts on both SPBs at the time of SPB duplication, but later on, at the metaphase to anaphase transition, it is found more concentrated on the bud-directed SPB [20,30,31]. However, a small fraction of Tem1 can still be found on the mother cell SPB also after anaphase [20,30]. Bub2/Bfa1 subcellular localization follows essentially the same pattern, with the notable exception that the complex completely disappears from the SPB remaining in the mother cell at the onset of anaphase [20,30,31]. Bub2 and Bfa1 are actually required for Tem1 SPB localization during interphase and mitosis, but not anymore in late anaphase [20]. Lte1 also shows a peculiar localization pattern, as it is confined in the bud (mostly at the bud cortex) throughout most of the cell cycle, spreading into the cytoplasm of both mother and daughter after the nucleus has migrated into the bud [20,30,31]. The spatial segregation of Lte1 and Tem1 has led to an attractive model that explains how nuclear division might be coupled with mitotic exit. According to this model, Tem1 is kept inactive at SPBs by Bub2/Bfa1 until the nucleus is pulled into the bud at the time of anaphase onset. Only in this case would Tem1 encounter its activator Lte1, thus leading to MEN activation and mitotic exit. Thanks to this spatial segregation, improper spindle and nuclear positioning would prevent Tem1 activation and delay mitotic exit and cytokinesis until errors are corrected, thus ensuring balanced chromosome partitioning between mother and daughter cell [20,31]. One problem with this model is, as already mentioned, that Lte1 is neither required for cell viability at physiological temperatures [28] nor for the unscheduled mitotic exit of mutants defective in spindle positioning [32]. Conversely, deletion of either *BUB2 *or *BFA1 *is sufficient to neutralize completely the spindle position checkpoint [20,31-33], suggesting that inactivation of the Bub2/Bfa1 complex might be the actual trigger of mitotic exit.

How might this be achieved? One mechanism that contributes to Bub2/Bfa1 inactivation is phosphorylation of both subunits by the polo kinase Cdc5 [34,35], although expression of an non-phosphorylatable form of Bfa1 does not cause a telophase arrest [34].

The current model for mitotic exit predicts that most of the regulation over mitotic exit takes place in the bud. Accordingly, the amount of Tem1 found on the bud-directed SPB roughly doubles during anaphase, when only small quantities of Tem1 can be found on the mother-bound SPB. However, Bub2/Bfa1 is also concentrated on the bud-directed SPB at the time the spindle passes through the bud neck; its levels drop dramatically only afterwards, in mid- to late-anaphase [30]. If penetration of the daughter-destined SPB into the bud has to signal mitotic exit [30], the simultaneous presence of Tem1 and Bub2/Bfa1 on that SPB during early anaphase is surprising and would appear as counter-productive for full Tem1 activation. Since Lte1 is required for Tem1 accumulation at the bud-directed SPB in anaphase [30], Lte1 localization to the bud cortex could serve as a backup mechanism that promotes mitotic exit in case alternative ways of activating Tem1 go wrong. This raises the question of how might Tem1 be activated primarily.

## Mother-bound control of the MEN

Recent data have highlighted an important role for the mother cell in controlling mitotic exit upon spindle mispositioning. This would seem quite logical in light of the fact that anaphase takes place within the mother cell when the spindle is not properly oriented [36,37]. In this case or when microtubules are depolymerised, Bub2/Bfa1 is retained on both SPBs, whereas the complex normally disappears from the mother-bound SPB as soon as the spindle elongates through the bud neck [30,38] (Fig. 2). Thus, the pool of Tem1 localized on the mother SPB could play a key role in setting the proper timing of MEN activation and be directly controlled by Bub2/Bfa1 and other colocalized regulators. Indeed, the myc-tagged Bub2 form described above and devoid of GAP activity persists on both SPBs throughout the cell cycle along with Bfa1 and Tem1, and prevents mitotic exit in a dominant fashion in cells expressing partially crippled Tem1, Cdc5 or Nud1. This suggests that disappearance of Bub2/Bfa1 from the mother-bound SPB contributes to timely mitotic exit [18]. Surprisingly, this form of Bub2 is not lethal for *lte1Δ *cells, suggesting that yet another mechanism participates in Tem1 activation at the end of mitosis. Importantly, the disappearance of Bub2 from the mother-bound SPB at the onset of anaphase depends on its GAP activity. Indeed, the Bub2^R85A ^mutant that lacks GAP activity remains constitutively at both SPBs despite being unable to recruit Bfa1, whereas myc-tagged Bub2 behaves similarly but does recruit Bfa1 [18]. Thus, Bub2 might have targets other than Tem1: in this scenario Bub2^R85A ^would have no GAP activity towards any of its substrates, explaining its inability to recruit Bfa1 at SPB and to activate the checkpoint, whereas myc-tagged Bub2 could be deficient only in stimulating the GTPase activity of Tem1. Hence, Bub2/Bfa1's GAP activity on Tem1 might be involved in regulating its subcellular localization rather than in triggering the checkpoint.

Disappearance of Bub2 from the mother-bound SPB in anaphase depends also on a functional septin ring and on several protein kinases localized at the bud neck [18], consistent with the notion that passage of one SPB through the neck signals mitotic exit [30]. Interestingly, perturbing the integrity of the septin ring can either lead to a lethal mitotic exit defect when the MEN is impaired, or cause improper mitotic exit in mutants defective for spindle positioning. While the former is in agreement with the septin ring function in allowing the release of Bub2/Bfa1 from the mother-bound SPB [18], the latter is Lte1-dependent and correlates with Lte1 spreading into the mother cell cytoplasm [39]. Thus, whether an altered septin ring causes delayed or accelerated mitotic exit depends on the balance between inactive and active Tem1 in the mother cell.

The Kin4 kinase, which prevents Cdc14 release from the nucleolus in response to defects in spindle alignment by antagonizing MEN activation, has been recently implicated in regulating Bub2/Bfa1 activity [40,41]. Although its precise function in the spindle position checkpoint is currently unknown, Kin4 seems to exert its inhibitory function through Bub2/Bfa1 and to counteract the Cdc5-dependent phosphorylation of Bub2 and Bfa1 [40,41]. Remarkably, Kin4 localizes at the cortex of the mother cell throughout most of the cell cycle (Fig. 2) and is found at the bud neck in late anaphase cells [40,41]. In addition, Kin4 is also detected on the mother-bound SPB between mid-anaphase until telophase, mirroring localization of Bub2/Bfa1. Importantly, in the presence of misoriented spindles Kin4 is localized on both SPBs along with the Bub2/Bfa1 complex [41] (Fig. 2). Thus, Kin4 could establish a domain of MEN inhibition within the mother cell. It is interesting to note that Kin4 displays some features in common with Dma1 and Dma2, two putative MEN inhibitors with high similarity to eachother [42] and homologous to *S. pombe *Dma1 [43] and human Chfr [44], which are both involved in mitotic checkpoints. Like for *KIN4*, deletion of *DMA1 *and *DMA2 *causes premature mitotic exit in the presence of misoriented spindles [42], but not when microtubules are depolymerized by spindle poisons [40-42]. Furthermore, overexpression of *DMA2*, like that of *KIN4*, delays mitotic exit by anatgonizing Tem1 activation [40,42]. It will be interesting in the future to investigate whether and how Dma1-2 and Kin4 regulate each other.

## Conclusion

So far, a definitive model for how yeast mitotic exit is controlled in response to spindle mispositioning has been hampered by the impossibility to monitor Tem1 activation individually at each subcellular location. Recruitment of the effector kinase Cdc15 to SPBs is assumed to be a good marker for Tem1 activation, but the timing of Cdc15 localization at each SPB is controversial [12,13,30,45-47]. In any case, it is becoming increasingly clear that overlapping mechanisms control the activity of this key GTPase, as depicted in Figure 2. When the spindle is mispositioned (Fig. 2, left side) Tem1 is kept inactive in the mother cell by Bub2/Bfa1 being localized on both SPBs and by the lack of exposure to Lte1 present in the bud. In these conditions, the Bub2/Bfa1 complex is maximally active due to the Kin4 kinase, which is present in the same cell compartment and counteracts the inhibitory phosphorylation of Bub2/Bfa1 by the polo kinase Cdc5. Transit of one SPB through the bud neck during proper anaphase (Fig. 2, right side) triggers the disappearance of Bub2/Bfa1 from the mother cell SPB through a mechanism involving Bub2 GAP activity and kinases localized at the bud neck with the septin ring. This likely contributes to activate Tem1 in the mother cell. Concomitantly, the pool of Tem1 on the daughter cell SPB gradually increases, with Lte1 contributing both to Tem1 enrichment and initial activation, perhaps in parallel with inhibitory phosphorylation of Bub2/Bfa1 by Cdc5. Late in anaphase, Tem1 activation is probably completed by the disappearance of Bub2/Bfa1 also from the daughter SPB, driving Cdc14 release from the nucleolus and mitotic exit. Thus, control over mitotic exit is emerging as being much more complex than initially thought. It will now be crucial to define which pool of Tem1 is relevant in promoting MEN activation and which of the many controls exerted on Tem1 is critical for coupling mitotic exit with proper spindle positioning. In addition, it will be important to address whether signal transduction cascades similar to MEN and SIN are regulated in response to spindle positioning defects in higher eukaryotic cells. Several homologues of MEN and SIN components have been found in animal and plant cells, although their role in controlling mitotic exit or cytokinesis is not always clear. In addition, centrosomes have been implicated in controlling cytokinesis in several systems [48,49] and anormalities in centrosome function have been linked to cancer development [50,51], suggesting that these organelles are key in ensuring maintenance of genome integrity.
